# Optimization of Solid Lipid Nanoparticle Formulation Using Design of Experiments, PART I: *Strategic Tool for the Determination of Critical Parameters Regarding Formulation and Process*

**DOI:** 10.3390/nano15131034

**Published:** 2025-07-03

**Authors:** Margot Cassayre, Dany Teles de Souza, Magalie Claeys-Bruno, Alexandre Altié, Philippe Piccerelle, Christophe Sauzet

**Affiliations:** 1 CNRS, IRD, IMBE, Avignon Université, Aix-Marseille University, 13385 Marseille, France; dany.souza@ifrj.edu.br (D.T.d.S.); m.claeys-bruno@univ-amu.fr (M.C.-B.); philippe.piccerelle@univ-amu.fr (P.P.); 2 CNRS, CINAM, Aix-Marseille University, 13009 Marseille, France; alexandre.altie@cnrs.fr

**Keywords:** solid lipid nanoparticle (SLN), optimization, formulation, physical characterization, design of experiments (DOE), statistics

## Abstract

This study presents a methodological framework for optimizing “blank” solid lipid nanoparticles (SLNs), focusing on the use of a design of experiments (DOE) approach. Rather than emphasizing the applications of SLNs, the objective is to identify and optimize critical formulation and process parameters—specifically those influencing particle size (PS), polydispersity index (PDI), and zeta potential (ZP)—during early development stages. A non-classical mixed design was applied using AZURAD^®^ software (version 4.4.1), incorporating a mixture variable for lipid composition (comprising carnauba wax, glyceryl behenate, glyceryl distearate), and two quantitative factors: the percentage of polysorbate 80 (P80) in the P80/sorbitan oleate surfactant system and ultrasound (US) treatment time. The DOE analysis identified P80 concentration as a key parameter, with optimal formulations observed when P80 ranged between 35% and 45%. A fixed P80 ratio of 41% and a US time of 7.5 min enabled precise adjustment of lipid composition. Following a desirability function analysis, an optimized formulation was obtained with a PS of 176.3 ± 2.78 nm, a PDI of 0.268 ± 0.022, and a ZP of −35.5 ± 0.36 mV. These findings validate the relevance of our DOE-based strategy, offering a scalable, cost-effective platform that reduces material use, time, and analytical effort in SLN development.

## 1. Introduction

In recent years, the development of solid lipid nanoparticles (SLNs) has gained considerable interest in the pharmaceutical and cosmetic fields due to their ability to enhance the solubility, stability, and bioavailability of active ingredients [1]. While their application potential, particularly for topical and transdermal delivery, has already been extensively addressed, including in our other publication [2], the focus of the present study lies elsewhere.

The goal of this article is to demonstrate how a design of experiments (DOE) approach can be used to develop SLN formulations in a statistically rigorous and resource-efficient manner. The objective is to demonstrate the value of DOE in identifying and optimizing critical formulation and process parameters that influence SLN quality, rather than exploring their biomedical or cosmetic applications.

SLN optimization largely depends on three parameters: particle size (PS), polydispersity index (PDI), and zeta potential (ZP). These parameters significantly affect formulation stability, drug release behavior, and bioavailability [2,3,4]. However, conventional trial-and-error methods used to optimize them are often time-consuming, costly, and poorly reproducible. Therefore, a structured and scalable methodology is needed to support more efficient development processes.

The primary objective of this study is to propose a resource-efficient methodology for the formulation of SLNs using a design of experiments (DOE) approach. Recent strategies use experimental designs (e.g., Box–Behnken used by N. Dudhipala [5]) to optimize independent variables like lipid content, surfactant/co-surfactant concentration, and sonication time [5,6]. Characterizing active-ingredient (AI)-loaded SLNs often requires advanced techniques and multiple collaborations (e.g., X-ray diffraction, differential scanning calorimetry, scanning/transmission electron microscopy), highlighting the need for a systematic, resource-efficient approach to formulation.

Unlike conventional methods, this study focuses on optimizing a blank SLN formulation—without incorporating an AI initially—to reduce production, analytical, and time-related costs, making the process more suitable for industrial applications.

Reduction of production costs: Developing SLNs often requires numerous formulation trials to achieve the desired characteristics (PS, PDI, and ZP). Conducting these trials directly with an AI can be prohibitively expensive, especially if the AI is costly. By first optimizing a blank SLN matrix, we can avoid the unnecessary use of expensive AIs during the initial screening phase.

Reduction of analytical costs: Characterizing SLNs typically involves expensive techniques such as X-ray diffraction (XRD), small-angle X-ray scattering (SAXS), wide-angle X-ray scattering (WAXS), and electron microscopy (TEM, SEM) [7]. By focusing on optimizing the blank SLN formulation based only on size, polydispersity, and zeta potential, we can minimize the need for extensive characterization techniques during the initial development stage. The full characterization will only be performed once the AI is incorporated, as required for stability studies and regulatory compliance.

Reduction of time and simplification of the screening process: Time is a critical factor in industrial R&D, and reducing the time spent on formulation can translate directly into cost savings. We chose ultrasound (US) as a screening method because it is quick, easy to implement, and operates via cavitation, similar to high-pressure homogenization (HPH), which is scalable for industrial production [8]. Additionally, industrial-grade ultrasound probes are available, making the process readily translatable to larger-scale manufacturing.

By employing a non-classical experimental design (mixte design), this study integrates three mixture variables and two quantitative parameters to optimize the SLN production:

• Mixture variables: Proportion of carnauba wax, glyceryl behenate, and glyceryl distearate.
• Quantitative parameters: The percentage of polysorbate 80 (P80) in the P80/sorbitan oleate (SO) ratio and the duration of US treatment.

These factors were selected based on their known influence on SLN characteristics [9,10,11] and preliminary testing to establish an optimal lipid-to-surfactant ratio.

This research aims to provide a robust, science-driven methodology for optimizing SLN formulations in a cost-effective manner, which can be adapted for both pharmaceutical and cosmetic applications. By focusing on experimental design and the identification of critical formulation and process parameters, this study provides an optimized blank SLN formulation that can be used as a flexible platform for the later incorporation of active ingredients [2]. This approach simplifies the development process and helps reduce the overall costs of SLN production and characterization. Our approach aligns with industrial needs by focusing on reducing time, material waste, and reliance on complex analytical techniques. The findings of this study hold the potential to accelerate the development of SLN-based products with enhanced performance, contributing to more efficient and sustainable production processes.

## 2. Materials and Methods

### 2.1. Materials

Carnauba wax (T-1) provided by IMCD France (Lyon, France), glyceryl behenate (Compritol 888 CG pellets), and glyceryl distearate (Precirol ATO) obtained from Gattefossé (Saint-Priest, France) were used as lipid materials. Polysorbate 80 (Tween 80) and sorbitan oleate (Span 80) obtained from Croda (Montigny-le-Bretonneux, France) were used as emulsifiers in the formulation.

### 2.2. Preparation of the SLNs

The aim of the study was to optimize the composition of the SLNs regarding PS, PDI, and ZP. These parameters are influenced by the process parameters such as the concentrations of surfactant and total lipids, the lipids-to-surfactant ratio, and the homogenization conditions; this is why we have kept the same parameters for all our experiments except for the ultrasound time [12].

In this investigation, we want to highlight the influence of the lipid and surfactant composition. To analyze these impacts and using preliminary tests, we decided to keep the proportions between the amount of lipids and emulsifier constant (1:2 ratio), and the PS, PDI, and ZP were evaluated.

The preparation contains:

- 5% of lipidic phase comprising carnauba wax (CW), glyceryl behenate (GB), and glyceryl distearate (GDS);
- 10% of surfactant: polysorbate 80 (P80) and co-surfactant: sorbitan oleate (SO).

The aqueous phase, consisting of a dispersion of P80 in water, was prepared under magnetic agitation and heated at 92 °C. The lipidic fraction: a mixture of CW, GB, GDS, and the second surfactant, the SO, was prepared through heating to 92 °C and mixed with a magnetic stirrer.

The hot aqueous phase containing the P80 was dispersed in the melted lipidic fraction through high-speed stirring using a T 25 ULTRA-TURRAX^®^ (IKA^®^—Werke GmbH and Co, KG, Staufen, Germany) at 10,000 rpm for 10 min at 92 °C ± 3 °C.

Then, the SLNs were produced by ultrasonication. The pre-emulsion formed was homogenized using sonication (Ultrasonics^TM^ Sonifier^TM^, SFX550, Fisher Scientific, Illkirch, France). The sonicator was used at 70% of amplitude for 1 to 10 min, depending on the formulation condition given by the DOE. The preparation was immediately cooled in an ice bath to obtain the SLNs.

Here are the different variables chosen to build the DOE (Table 1):

- For the aqueous phase:
  • X_1_: Percentage of P80 in surfactant and co-surfactant amount (P80/SO), variation domain 0–100% of surfactant in surfactant and co-surfactant amount. For example, if there is 100% of P80, it means that we will have 0% of SO or if there is 50% of P80, we will have 50% of SO.
- For the sonication conditions:
  • X_2_: Ultrasound time (US time), variation domain from 1 to 10 min;
- The lipidic phase composition is expressed as a proportion of each component in the mixture, representing 5% of the final formula;
  • X_3_: Carnauba wax, variation domain from 0 to 1;
  • X_4_: Glyceryl behenate, variation domain from 0 to 1;
  • X_5_: Glyceryl distearate, variation domain from 0 to 1.

### 2.3. Analysis of Particle Size (PS) and Polydispersity Index (PDI)

All the measurements were performed in triplicate using a Malver Zetasizer Nano ZS90 (Malvern Instruments Ltd., Worcestershire, UK) at a temperature of 25 ± 2 °C (sensitivity range of measurement: 0.3 nm–5 µm). The mean particle size (z-average diameter) and PDI were measured using the dynamic light scattering (DLS) technique and were obtained by averaging the measurements at an angle of 90°.

Prior to the measurement, all samples were diluted with distilled water (1/100) to obtain a suitable scattering intensity.

### 2.4. Zeta Potential (ZP) Analysis

The ZP of SLNs was also determined using the Malver Zetasizer Nano ZS90 (Malvern Instruments Ltd., Worcestershire, UK) at a temperature of 25 ± 2 °C. This device uses a combination of two measurement techniques: electrophoresis and laser Doppler to measure the zeta potential by determining the electrophoretic mobility of the particles. It indicates the prediction of the physical stability of the SLNs. Before the measurement, each sample was diluted in distilled water (1/60), and the results were determined by averaging the twenty measurements. All measurements were performed in triplicate.

### 2.5. Design of Experiments

Design of experiments (DOE) [6,10,11,13] is a statistical method that provides specific information for the selection and order of experiments. They enable us to identify the quantitative influence of chosen parameters by investigating the responses of the experiment. They involve performing a limited number of experiments, where combinations are chosen to study the influence of parameters (factors). This is a useful approach to assess adequate conditions, in our case, leading to a particle size around 100 nm, the smallest possible polydispersity index, and the highest zeta potential in absolute value. For this purpose, results from a set of experiments were used to establish predictive models for each investigated response. According to the literature and as discussed in the introduction, five factors were chosen: two quantitative factors and three mixture factors for the waxy phase as described previously.

The factors are presented with their respective variation domains in Table 1.

To establish a relationship between the measured responses and the factors, polynomial models were used. A mixed model combining the model for quantitative factors and the model for the mixture factors was set.

The two quantitative factors, the percentage of P80 in surfactant and co-surfactant couple (P80/SO) and time of US, were studied using a quadratic model, as it allows us to consider the potential curvature and/or torsion of the surfaces in the domain. The model used is presented in Equation (1).

(1) $$yquanti=\beta_{0}+\beta_{1}X_{1}+\beta_{2}X_{2}+\beta_{11}X_{1}^{2}+\beta_{22}X_{2}^{2}+\beta_{12}X_{1}X_{2}$$

*X*_i_ are the coded values of variables, *β*_0_ is the intercept term, *β*_i_ are linear coefficients for the percentage of P80 and time of US, and with *β*_ii_ and *β*_ij_ their squared effects and interaction effects.

The mixture factors were studied using a reduced cubic Scheffé Model and presented in Equation (2):

(2) $$ymixture=\beta_{3}X_{3}+\beta_{4}X_{4}+\beta_{5}X_{5}+\beta_{34}X_{3}X_{4}+\beta_{35}X_{3}X_{5}+\beta_{45}X_{4}X_{5}+\beta_{345}X_{3}X_{4}X_{5}$$

*X*_3_, *X*_4_, and *X*_5_ represented the levels of three constituents, and *β*_i_, *β*_j_, and *β*_k_ were regression coefficients.

Finally, the mathematical model chosen for the design was a multiplicative model reduced to degree 3 coefficients. Its mathematical expression is presented in Equation (3). Its purpose is to explore the effects of the mixture and quantitative parameters while considering interactions between both types of parameters.

(3) $$\begin{array}{c} Y=\beta_{3}X_{3}+\beta_{4}X_{4}+\beta_{5}X_{5}+\beta_{34}X_{3}X_{4}+\beta_{35}X_{3}X_{5}+\beta_{45}X_{4}X_{5}+\beta_{345}X_{3}X_{4}X_{5}+\\ \beta_{13}X_{1}X_{3}+\beta_{14}X_{1}X_{4}+\beta_{15}X_{1}X_{5}+\beta_{134}X_{1}X_{3}X_{4}+\beta_{135}X_{1}X_{3}X_{5}+\beta_{145}X_{1}X_{4}X_{5}+\\ \beta_{23}X_{2}X_{3}+\beta_{24}X_{2}X_{4}+\beta_{25}X_{2}X_{5}+\beta_{234}X_{2}X_{3}X_{4}+\beta_{235}X_{2}X_{3}X_{5}+\beta_{245}X_{2}X_{4}X_{5}+\\ \beta_{1-1\;3}X_{1}^{2}X_{3}+\beta_{1-1\;4}X_{1}^{2}X_{4}+\beta_{1-1\;5}X_{1}^{2}X_{5}+\beta_{2-2\;3}X_{2}^{2}X_{3}+\beta_{2-2\;4}X_{2}^{2}X_{4}+\beta_{2-2\;5}X_{2}^{2}X_{5}+\\ \beta_{123}X_{1}X_{2}X_{3}+\beta_{124}X_{1}X_{2}X_{4}+\beta_{125}X_{1}X_{2}X_{5}+ei \end{array}$$

where *Y* was the studied responses (PS, PDI, and ZP), and *ei* was the experimental error.

To evaluate the coefficients of this method, a set of 63 experiments was selected using an exchange algorithm based on D-optimality criteria [9,14] since a “classical” design of experiments does not apply to our system (due to the combination of the mixture and quantitative variables). Experiments 51, 52, and 55 were repeated twice to calculate the experimental variance and reinforce the reliability of the final model validations. (See Table 2).

**Figure 1:**
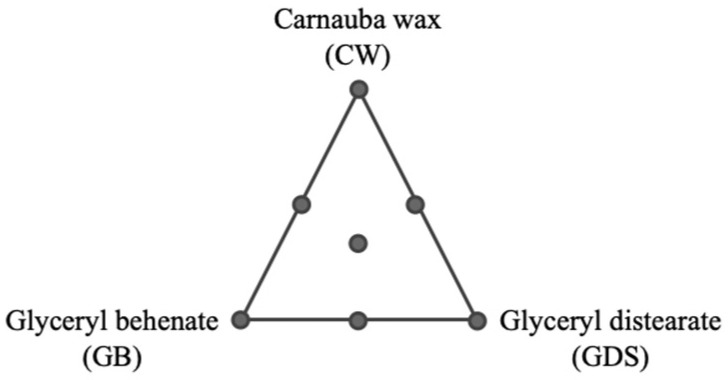
Representation of the seven experimental conditions established for the mixture design with three mixture variables: proportion of carnauba wax, proportion of glyceryl behenate, and proportion of glyceryl distearate in the lipidic fraction.

**Figure 2:**
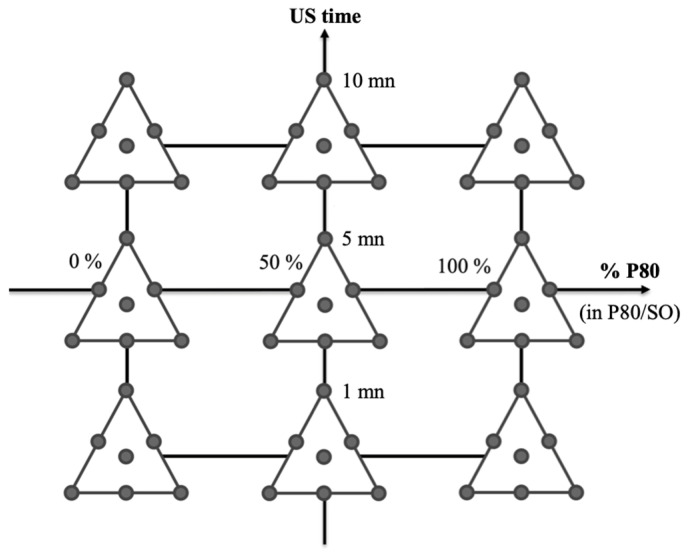
Representation of the 63 experimental conditions established for the design of experiments combining the two quantitative variables (% of polysorbate 80 in P80/SO couple and US time) and the mixture design with three mixture variables (Figure 1).

In order to explain the design construction, we can represent the experimental conditions for the mixture variables through a ternary plot with seven experiments (Figure 1). These mixtures were tested for the nine conditions of the quantitative variables, leading to 63 experiments (Figure 2).

### 2.6. Optimization Using Desirability Functions

One of the statistical methods that can be used to determine the optimized formulation is the desirability function approach. This tool involves transforming the Y_i_ responses into a D function, known as the desirability function, which is defined in a specific range of the Y_i_ variation using AZURAD^®^ software(version 4.4.1). Desirability values were established for each of the individual responses, and all the responses were associated with their own desirability function *d_i_*, which varies from 0% to 100% depending on the closeness of the target response. When the desirability is equal to 0% it coincides with the point that is furthest from the target value, while that equal to 100% coincides with the point closest to the target value. To calculate the global desirability, the following equation was used:

(4) $$D=({d_{1}}^{w_{1}}\times {d_{2}}^{w_{2}}\times {d_{3}}^{w_{3}})\frac{1}{\sum wi}$$

*d*_i_: partial desirability function;

*w*_i_: weight assigned to answer I;

The type, coefficient, and weight of each response were summarized in Table 3.

### 2.7. Differential Scanning Calorimetry (DSC) Analysis

DSC was performed to characterize the thermal behavior and the physical state of the lipids and blank SLN by using a DSC 25 instrument (TA Instrument, New Castle, DE, USA). The nanoparticles were lyophilized using an Alpha 1–4 LCS (Christ, Fisher Scientific, Illkirch-Graffenstaden, France). Then, 50 mL of SLNs were placed in a 100 mL glass bottle. The entire bottle containing the nanoparticle preparations was placed at −25 °C overnight. To be freeze-dried, the samples underwent an initial primary desiccation phase with a condenser temperature of −55 °C and a vacuum set at 1.030 mbar, followed by a secondary desiccation phase lasting approximately 6 h with a plateau temperature set to 30 °C and a maximum vacuum of 0.0054 mbar.

Briefly, 5–7 mg of samples were placed in a hermetic aluminum pan. The system was calibrated using an indium standard, and the sample was run against a hermetic empty reference pan. The analyses were performed from 20 °C to 120 °C at a heating rate of 5 °C min^−1^ by flushing with nitrogen at a rate of 80 mL/min to obtain an inert gas atmosphere in the DSC cell. TRIOS software (version 5.1) was used to obtain the DSC thermograms.

### 2.8. Scanning Electron Microscopy (SEM) Analysis

The morphology of the nanoparticles was evaluated using the scanning electron mi- croscopy (SEM) technique. A JEOL JSM-7900F microscope (Tokyo, Japan) was used. Briefly, a small amount of diluted blank SLN was placed on a silicium wafer support attached to a stub and placed under vacuum for several minutes to dry the sample using a turbomolecular pump. SEM was operated in standard mode (SEM) at 5 kV (WD = 11 mm using chamber secondary electron detector) and using 2 kV decelerating GB (gentle beam) mode for a final accelerating voltage of 1 kV (WD = 4 mm using an Everhart-Thornlet secondary electron detector), which is a specific mode to analyze isolated materials without carbon deposition. All analyses were performed at room temperature (20 °C). Digital images were acquired using already owned JEOL SEM-PC software (version 3.0).

## 3. Results and Discussion

### 3.1. Data Analysis

The analysis of the data obtained for the 63 + 3 replicate experiments was performed by AZURAD^®^ software, using a statistical approach at first for a global data evaluation. Then, based on the experimental results, the estimation of the model coefficients (Equation (3)) were calculated using multi-linear regression. To validate the postulated model, an ANOVA test and a regression test were used. The models’ adequacy and significance were checked for each selected response. The significance and accuracy of the model were then validated by ANOVA (Analysis of variance) (*p*-value and R^2^). The regression is significant as the *p* value (%) for the three responses, PS, PDI, and ZP was, respectively, 0.0005, 0.4199, and ≤0.0000, and the R^2^ was, respectively, 0.80, 0.67, and 0.98. For each model, predictive values were calculated in the entire domain of interest and compared to experimental responses. The models were proven to be accurate if the theoretical responses corroborated with experimental responses.

### 3.2. Analysis of Particle Size (PS)

**Figure 3:**
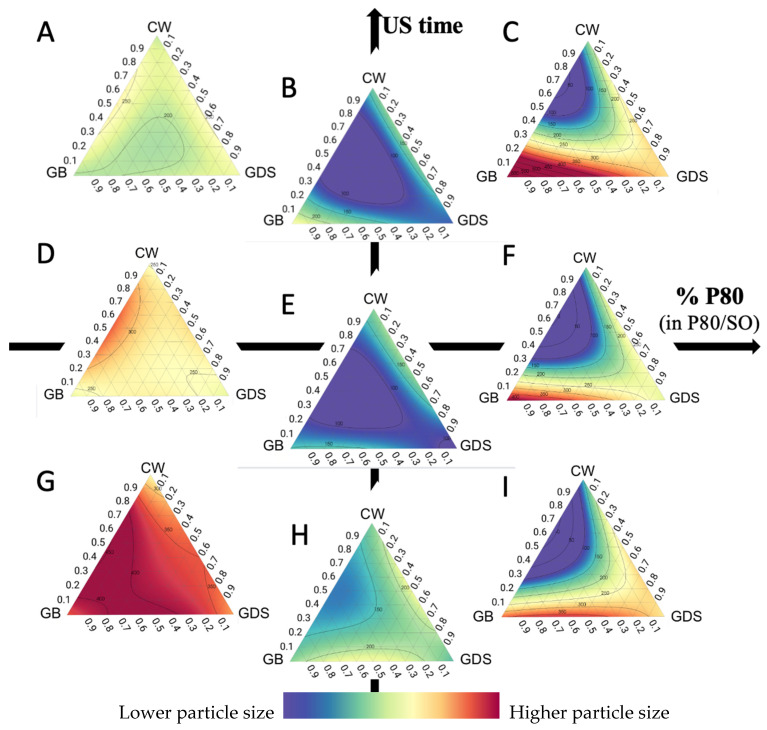
Ternary plot representations of the particle size as a function of the percentage of polysorbate 80 (in P80/SO): ultrasound time (min). (**A**) 0:10; (**B**) 50:10; (**C**) 100:10; (**D**) 0:5; (**E**) 50:5; (**F**) 100:5; (**G**) 0:1; (**H**) 50:1; (**I**) 100:1.

Taking into account the particle-size (PS) analyses, we can observe that in the presence of SO only (Figure 3A,D,G), it is necessary to increase the ultrasound time to achieve a better PS, regardless of lipid composition, and that it remains difficult to achieve a good PS.

On the other hand, a small PS mainly requires a mixture of P80/SO (Figure 3B,E,H). The presence of P80 probably allows a better arrangement of the surfactants on the surface of the submicronic particles, which allows a reduction in their size [3].

However, the more the amount of P80 increases, the more the reduction in size becomes dependent on the lipid composition. Indeed, the presence of P80 has an impact on the lipid composition and, in particular, on the percentage of CW (Figure 3C,F,I). In the presence of P80, a minimum of 30% CW is required to achieve a suitable PS around 100 nm.

We also observe that isoreponse curves are much closer to each other when we only have P80, which means the slightest change in lipid composition will have a fairly significant impact on particle size (Figure 3C,F,I). On the other hand, when the quantity of SO is increased, the isoreponse curves move apart, thus leaving higher margins of error in lipid composition (Figure 3B,E,H). CW has a second impact on the size of the particles; in fact, the more CW is added, the less the presence of P80 has an impact on size.

**Figure 4:**
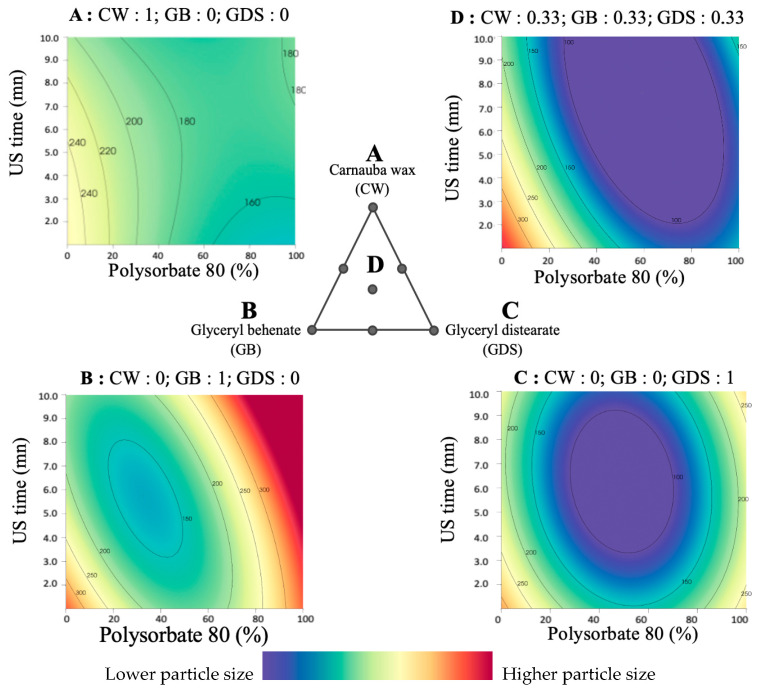
Surface plot representations of the particle size as a function of the percentage of polysorbate 80 (in P80/SO) and ultrasound time in four places of the ternary diagram. (**A**) with carnauba wax (CW) as the sole lipid (proportion = 1); (**B**) with glyceryl behenate (GB) as the sole lipid (proportion = 1); (**C**) with glyceryl distearate (GDS) as the sole lipid (proportion = 1); (**D**) with an equal mixture of CW, GB, and GDS (proportion = 0.33 each).

Our findings indicate that achieving a particle size approaching 100 nm necessitates the use of a ternary mixture, regardless of ultrasound exposure time, and requires a P80 concentration exceeding 30% (Figure 4D). Conversely, obtaining a PS close to 100 nm is unattainable using solely GB or CW (Figure 4A,B). On the other hand, Precirol alone can be combined with a 50/50 mixture of P80 and SO to successfully attain the desired particle size (Figure 4C).

### 3.3. Analysis of Polydispersity Index (PDI)

**Figure 5:**
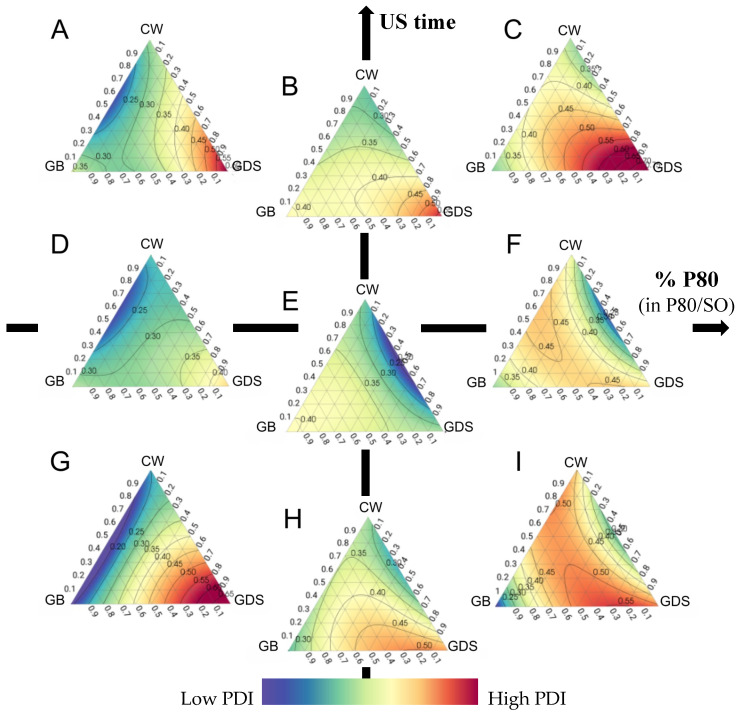
Ternary plot representations of the polydispersity index as a function of the percentage of polysorbate 80 (in P80/SO): ultrasound time (min). (**A**) 0:10; (**B**) 50:10; (**C**) 100:10; (**D**) 0:5; (**E**) 50:5; (**F**) 100:5; (**G**) 0:1 (**H**) 50:1; (**I**) 100:1.

**Figure 6:**
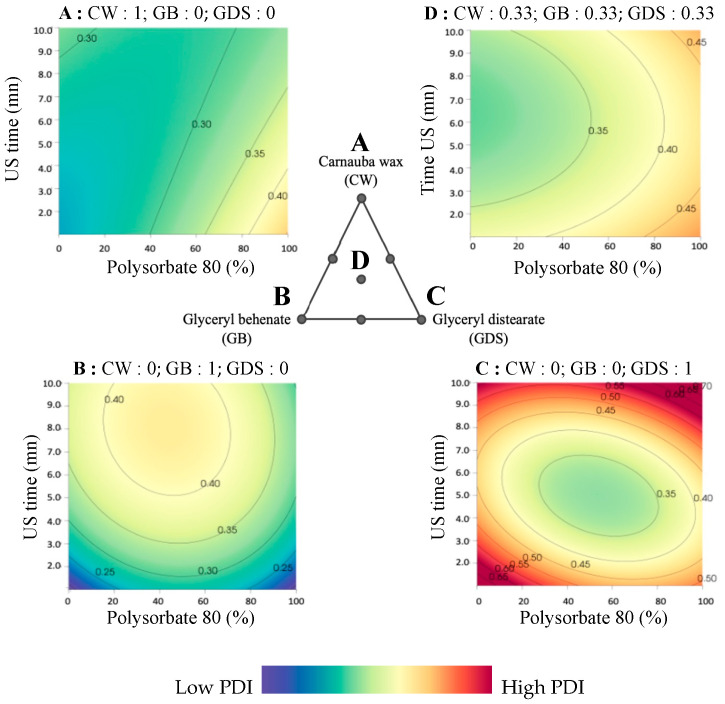
Surface plot representations of the polydispersity index as a function of the percentage of polysorbate 80 (in P80/SO) and ultrasound time in four places of the ternary diagram. (**A**) with carnauba wax (CW) as the sole lipid (proportion = 1); (**B**) with glyceryl behenate (GB) as the sole lipid (proportion = 1); (**C**) with glyceryl distearate (GDS) as the sole lipid (proportion = 1); (**D**) with an equal mixture of CW, GB, and GDS (proportion = 0.33 each).

Regarding the Polydispersity Index (PDI), it is evident that the ultrasound exposure duration exerts a discernible influence on this parameter. Achieving an optimal PDI entails adjusting the lipid composition in accordance with the proportions of P80 and SO present in the formulation. However, in our conditions, the acceptable zones for the PDI are very limited (Figure 5 and Figure 6).

Specifically, when the formulation consists of 100% SO (so 0% of P80), ultrasound duration has a modest effect, requiring the use of a binary mixture comprising GB and CW (Figure 5A,D,G).

Conversely, when employing a 50/50 mixture of P80 and SO, it is advisable to opt for a combination with GDS and CW. In such instances, an ultrasound exposure time of 5 min is recommended. Indeed, as can be seen in Figure 5B,E,H, a too short or too long sonication time can lead to a higher PDI. High pressure and turbulence produced by increasing the sonication time lead to agglomeration and fusion of the submicronic particles in the dispersed phase, generating populations of different sizes and consequently an increase in the PDI [15,16]. On the contrary, a shorter sonication time reduces shear and turbulence, which prevents particle size from decreasing, forming particles with various sizes, and leading to an increase in PDI [15].

Lastly, when the formulation exclusively comprises P80, irrespective of ultrasound time and lipid composition, the attainment of a favorable PDI remains challenging (Figure 5C,F,I).

As previously noted, the lipid composition significantly influences the Polydispersity Index (PDI). CW exhibits a reduced sensitivity to ultrasound duration compared to other lipid compounds, with surfactant composition playing a pivotal role. A higher proportion of SO coincides with improved PDI results, however, without ever reaching an acceptable PDI (Figure 6A). In the case of GB as the exclusive lipid (Figure 6B), a shorter or longer ultrasound duration correlates with improved PDI outcomes, but as previously noted, these conditions aren’t size-appropriate. Indeed, it is possible to obtain a good repartition of particles without reaching a good size. When considering GDS as the unique lipid component, achieving a favorable PDI proves challenging (Figure 6C). However, it is important to note that US time and percentage P80 play an essential role in increasing the PDI with this lipid, and the same conditions (US time around 5 min and a mixture of 50/50% of P80/SO) are required to achieve a good size.

Finally, when employing a lipid ternary mixture, surfactant composition and ultrasound duration are critical in achieving a desirable PDI. It is imperative to maintain a minimum SO ratio of 50%, accompanied by an ultrasound duration of 3 min or more to optimize PDI, but it remains difficult to obtain a good PDI (Figure 6D).

### 3.4. Zeta Potential (ZP) Analysis

Following an in-depth analysis of various formulations through experimental design, our observations reveal that P80 and SO exert a substantial influence on the zeta potential, while the lipid composition itself only has a minor impact. This finding corroborates the findings of Mosqueria et al., who reported that the nature of the oil core does not significantly alter the zeta potential value. They attributed this phenomenon to the complete encapsulation of the lipid within the polymer matrix, rather than its localization at the particle interface [17].

Significant positive or negative zeta potential values play a crucial role in maintaining stable colloidal suspensions, as the large repulsive forces effectively hinder particle aggregation by counteracting the potential agglomeration resulting from inadvertent interactions among neighboring submicronic particles [18].

**Figure 7:**
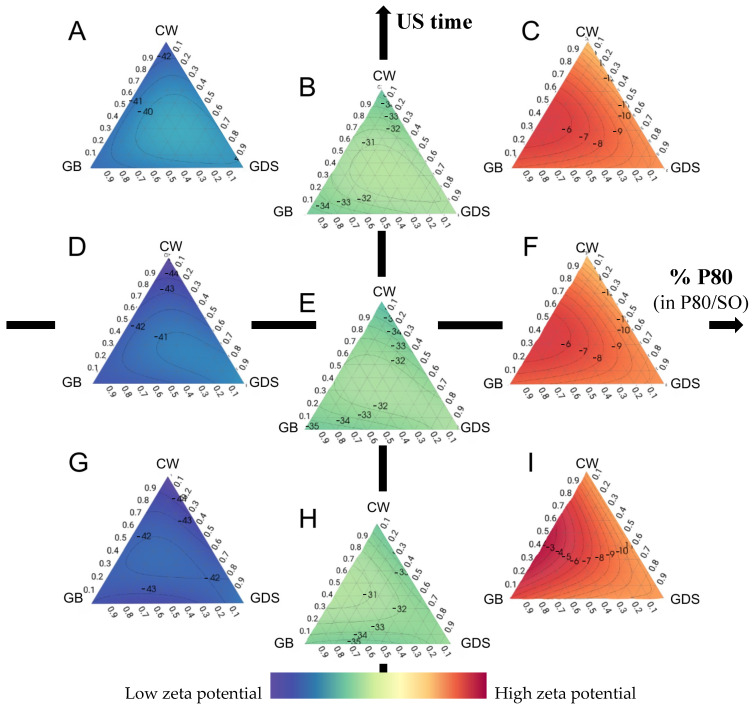
Ternaries plot representations of the zeta potential as a function of the percentage of polysorbate 80 (in P80/SO): ultrasound time (min). (**A**) 0:10; (**B**) 50:10; (**C**) 100:10; (**D**) 0:5; (**E**) 50:5; (**F**) 100:5; (**G**) 0:1 (**H**) 50:1; (**I**) 100:1.

In our investigation, when utilizing 100% P80 as the surfactant, the zeta potential exhibited a minimal range, typically ranging from −3 to −12 mV (Figure 7A,D,G). In this scenario, it is important to note that with an increase in the zeta potential, a corresponding improvement in the Polydispersity Index (PDI) is observed, particularly in the presence of P80 alone. This observation implies that lipid composition exerts a relatively modest influence on the optimization of the zeta potential, and it is partially linked to the PDI.

On the other hand, the inclusion of SO consistently yielded the most favorable zeta potential results (from −28.73 to −47.5 mV), independently of variations in lipid composition and ultrasound exposure duration (Figure 7B,C,E,F,H,I).

Several hypotheses can explain this phenomenon. Indeed, the presence of a negative charge surrounding the particles could be attributed to the residual electrolytes resulting from the ethoxylation catalyst of non-ionic surfactants such as SO [19].

Another hypothesis is that the adsorption of impurities or OH species from water to the O/W interface potentially results in the imposition of negative charges onto the droplets and could explain the higher zeta potential with SO [20].

Among these hypotheses, Rubiano et al. [21], who used the same surfactant couple as we did, showed that the employed stabilizer surfactants, namely Tween 80 and Span 80, are characterized as being electrically neutral, and hence, the anticipated values of ZP should be close to zero. Nonetheless, it is noteworthy that all the obtained zeta potential values showed a negative charge. These findings could be explained by the autoprotolysis of water, which spontaneously formed a thin monolayer of hydroxyl ions at the interface between the wax–surfactant–water [22,23]. Moreover, the electrical conductivity can be attributed to the existence of hydronium and hydroxyl ions originating from the autoprotolysis of water and the limited quantities of carbonic acid that may form spontaneously through the interaction of CO_2_ with the dispersing aqueous medium. Indeed, as the waxy phase is dispersed in the aqueous phase using an Ultra-Turrax, this process may introduce a small number of air bubbles containing CO_2_ (g), which subsequently convert into carbonic acid upon contact with water [21,24].

Finally, the presence of SO at the particle surface would result in enhanced electrostatic stabilization and reduce the aggregation of particles compared to P80. This could be attributed to the formation of a more substantial electrical double layer forming a protective layer at the particle surface, which reduced the Van der Waals interactions and aggregation, thus increasing the zeta potential [25].

On the other hand, as mentioned earlier, SO alone does not yield favorable particle sizes. Therefore, it becomes imperative to make a mixture of P80 and SO to strike a balance between particle size and zeta potential.

**Figure 8:**
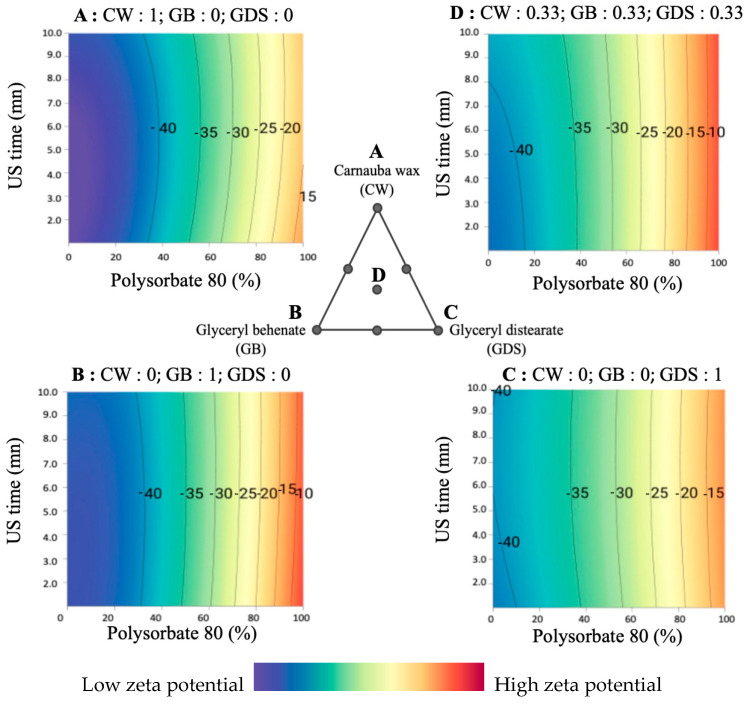
Surface plot representations of the zeta potential as a function of the percentage of polysorbate 80 and ultrasound time in four places of the ternary diagram. (**A**) with carnauba wax (CW) as the sole lipid (proportion = 1); (**B**) with glyceryl behenate (GB) as the sole lipid (proportion = 1); (**C**) with glyceryl distearate (GDS) as the sole lipid (proportion = 1); (**D**) with an equal mixture of CW, GB, and GDS (proportion = 0.33 each).

Here (Figure 8), we present a comprehensive illustration of the influence of surfactant composition. It is feasible to incorporate a maximum of 30% P80 while maintaining a favorable zeta potential.

Furthermore, the parallel nature of the isoresponse curves with respect to ultrasound duration signifies that it exerts no discernible influence on the zeta potential.

On the other hand, we notice a slight impact of the lipid composition. Indeed, CW and GB allow for obtaining a better zeta potential (Figure 8A,B) than the GDS or the ternary mixture (Figure 8C,D).

In summary, these zeta potential results can be explained by considering that a reorganization of the electrical double layer in the dispersed particles occurs over time. Therefore, the ions located in this layer move toward the bulk, acquiring better electrical mobility; in our study, this phenomenon is induced by the amount of P80/S0.

### 3.5. Desirability and Optimum Formulation

The experimental design combining a mixture design (with three variables) and two quantitative variables was followed by subsequent optimization of the three responses through the use of a desirability function approach [26]. The desirability function involves the transformation of the three responses Y_1_, Y_2_, and Y_3_ into individual desirability d_1_, d_2_, and d_3_, respectively.

The criteria for the optimization of the five variables X_1_, X_2_, X_3_, X_4_, and X_5_ were set in ranges, and goals were defined for each response.

Using the desirability function, with all the selected goals, the optimization procedure was carried out to obtain the surface response.

**Figure 9:**
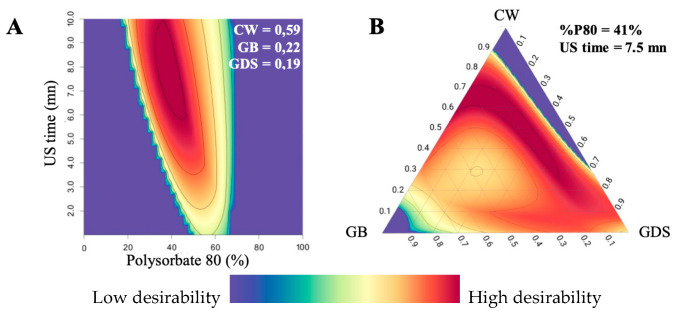
Representation of (**A**) surface plot global desirability with a proportion of CW = 0.59; GB = 0.22; GDS = 0.19, and (**B**) ternary plot global desirability with 40% P80 (in P80/SO couple) and 5.5 min of US time.

In our case, the global desirability is represented by Figure 9. With the surface plot representation (Figure 9A), we noticed that the percentage of P80 needs to be between 35–45%, and the US time higher than 6 min to obtain a high desirability (area in red). With these parameters, the range of the lipids should be: 0.25 to 0.7 for CW, 0.1 to 0.4 for GB, and 0 to 0.6 for GDS (Figure 9B).

Consequently, with the obtained desirability value, an optimized formulation (OP), was obtained in the best desirability area in red. In the blue area, desirability D is equal to 0%, meaning that at least one DOE response is not satisfied. The OP formulation was formulated to verify the predictive capacity of the model. The specific variable values are presented in Table 4. Subsequently, the same responses, namely particle size, polydispersity index (PDI), and zeta potential, were assessed for this formulation and compared with the predicted values, as outlined in Table 4.

Upon juxtaposing the predicted values with the experimental outcomes, disparities were identified, in particular regarding particle size. Indeed, the measurements obtained from Zetasizer analyses exceeded the predicted one, generated by the models. These results can be elucidated by the model error for the particle size and the error of the Zetasizer. However, even if the particle size is slightly larger than the initially predicted values, it still falls within the interval boundaries fixed at the beginning (50 to 200 nm). Conversely, in the case of the polydispersity index (PDI) and zeta potential, only minor disparities were noted between the predicted and observed values. Moreover, the observed values are slightly better than the predicted one. Despite the disparities observed in terms of particle size, these results can be explained by the close dichotomic relationship between the SO proportion and the P80 amount. Indeed, we have to maximize the SO part in order to obtain a good zeta potential and, conversely, minimize the SO to attain a smaller particle size.

Thanks to the experimental design, we successfully identified an optimal formulation for blank SLN using minimal resources, using solely the ZetaSizer. However, now that we have an optimized formulation, it is crucial to confirm the successful formation of SLN, particularly through differential scanning calorimetry (DSC) analysis and scanning electron microscopy (SEM).

### 3.6. Differential Scanning Calorimetry (DSC)

**Figure 10:**
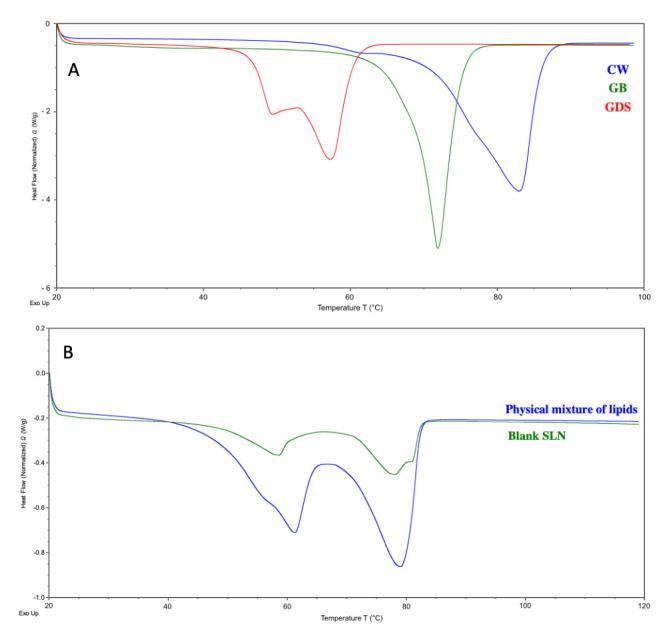
DSC thermograms of (**A**) the raw materials, CW (blue), GB (green), and GDS (red), and (**B**) the physical mixture of lipids (blue) and the blank SLN (green).

The data from the DSC analysis are presented in Table 5. The DSC curves were analyzed and endothermic peaks were observed at 82.96 °C, 71.91 °C, and 57.25 °C with a shoulder at 49.39 °C, for CW, GB and GDS respectively (Figure 10). The results were found to be appropriate compared to the literature [12,27,28]

The physical mixture of lipids (in the same proportions as the blank SLN) exhibits two endothermic thermal events. The first, corresponding to the melting of the GB and GDS mixture, has an onset of 50.76 °C with a peak temperature at 61.13 °C. The second, associated with CW melting, has an onset of 70.47 °C and a peak temperature at 79.19 °C.

Similar thermal events are observed for the blank SLN, with two melting transitions corresponding to the GB and GDS mixture (onset at 50.10 °C, peak at 58.45 °C) and CW (onset at 71.08 °C, peak at 78.20 °C).

The melting enthalpy of the first endothermic peak is higher in the lipid mix (36.438 J/g) than in the blank SLN (10.081 J/g). Similarly, the melting enthalpy of CW decreases from 52.159 J/g in the lipid mix to 18.711 J/g in the blank SLN.

The observed decrease in onset and peak melting temperatures, accompanied by a reduction in melting enthalpy, confirms the formation of solid lipid nanoparticles (SLNs). This phenomenon can be explained by a combination of physical and thermodynamic factors. As particle size decreases, the surface area-to-volume ratio increases significantly, leading to enhanced surface Gibbs free energy and higher chemical potential [29]. According to the Kelvin effect, this elevation in surface energy facilitates melting at lower temperatures, as less energy is required to overcome intermolecular lattice forces compared to bulk lipid materials [30,31]. Together, these thermal behaviors are consistent with the formation of submicron lipid particles.

As this study focused on the formulation of “blank” SLNs (without AI), no interaction between the lipid matrix and an AI was assessed. However, in our subsequent study, where active was incorporated into these optimized blank SLNs, the encapsulated compound was found in an amorphous state, and the overall physicochemical stability of the SLNs was maintained [2].

### 3.7. Scanning Electron Microscopy (SEM)

To gain further insights into particle size and morphology, SEM analysis was conducted.

**Figure 11:**
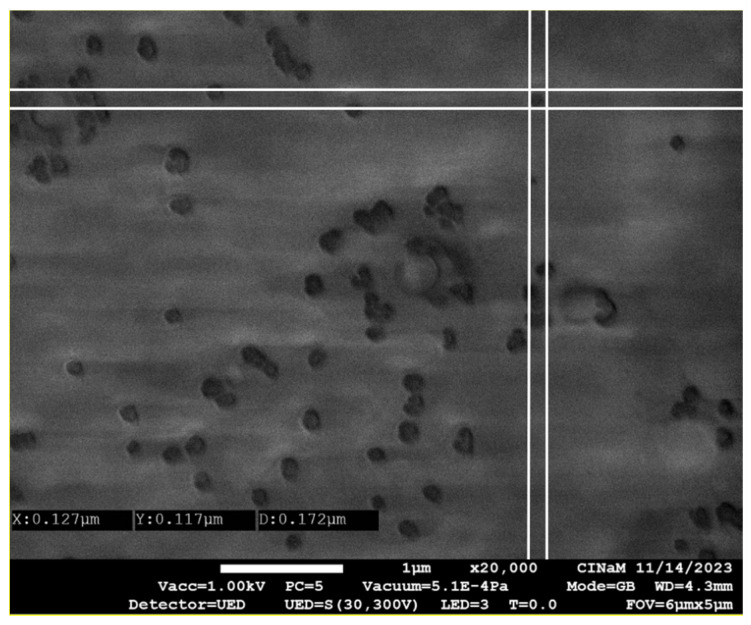
Size distribution of optimized blank SLN by scanning electron microscopy (SEM) × 20,000.

Figure 11 presents a microscopic image of the optimized blank SLN, revealing that most particles exhibit a spherical shape. However, some deviations from this morphology are observed, likely due to lipid deformation occurring during the drying process. Additionally, the size distribution of the optimized blank SLN appears homogeneous, with an average size of approximately 172 µm, as shown in Figure 11. This measurement is consistent with the particle size obtained from DLS analysis, further confirming the results.

### 3.8. Focus on DOE Interest in SLN Formulation

This study highlights the relevance and efficiency of DOE as a strategic tool to optimize SLN formulations, focusing on key physicochemical parameters such as particle size, PDI, and zeta potential. Although an initial set of 63 experiments was conducted, the DOE approach enabled robust modeling of formulation variables (namely lipid composition, surfactant ratio, and ultrasound duration), leading to the identification of optimal regions for desired characteristics. By identifying optimal desirability regions, we significantly reduced the number of trials required upon active compound incorporation. Only eight formulations were needed to identify a stable system with high encapsulation efficiency [2]. This also allowed us to assess the impact of the active compound on critical parameters such as size, PDI, and zeta potential. This represents a significant reduction in experimental workload compared to a conventional trial-and-error strategy.

Importantly, the developed SLN platform demonstrates sufficient flexibility to accommodate different application goals: nanoparticles below 100 nm for pharmaceutical delivery requiring systemic or to deliver medications to precise target locations within the body [32], and those above 200 nm for cosmetic formulations favoring localized release, Adhesiveness, occlusion and skin hydration [33,34]. The methodology confirms that DOE not only enhances process understanding and reproducibility, but also supports rational formulation development across various domains [35,36]. Moreover, this approach may be extended to other nanocarrier systems, offering a predictive framework to accelerate formulation screening while conserving resources.

## 4. Conclusions

This study presents a comprehensive exploration of “blanks” SLNs prepared using the ultrasonication method and optimized through the innovative application of a new experimental design approach. This preliminary step provides crucial information about the parameter in SLN production. We employed a multiplicative model that integrates two quantitative variables (percentage of polysorbate 80 and ultrasonication time) and three mixture variables (proportion of carnauba wax, proportion of glyceryl behenate, and proportion of glyceryl distearate).

The results highlighted the substantial influence of both formulation composition and process parameters on SLN-particle size, polydispersity index, and zeta potential. Notably, while there was a slight deviation between predicted and observed values, the obtained particle sizes of the optimized formulation remained within acceptable ranges for cosmetic SLNs. It can be seen that the model used to optimize the formulation of SLNs is fairly well adapted.

Moving forward, this article lays the foundation for the development of formulations by enhancing product robustness, reducing production costs, and accelerating time to market. Further investigations will focus on assessing the encapsulation efficiency, skin penetration, and stability of the SLN formulations, with regard to the impact of additive incorporation on overall stability. The next step involves the domain exploration around this optimized SLN formulation with active ingredients encapsulated inside [2].

This next study represents a pivotal aspect for cosmetic applications. These endeavors will collectively advance our understanding of SLNs as potential carriers for cosmetic formulations and contribute to their broader applicability in the field of pharmaceutical and cosmetic sciences.

## Figures and Tables

**Table 1:** Studied factors with their variation domains: for *X*_1_, variation domain expressed as a percentage of surfactant and co-surfactant amount representing 10% of the final composition; for *X*_3_ to *X*_5_, variation domain expressed from 0 to 1 representing 5% of the final formulation [2].

Variable	Factors	Nature of the Factor	Variation Domains
X_1_	Percentage of polysorbate 80 (P80) in P80/SO couple	Quantitative variable	0 to 100%
X_2_	US time	Quantitative variable	1 to 10 min
X_3_	Proportion of Carnauba wax (CW)	Mixture variable	0 to 1
X_4_	Proportion of Glyceryl behenate (GB)	Mixture variable	0 to 1
X_5_	Proportion of Glyceryl distearate (GDS)	Mixture variable	0 to 1

**Table 2:** The 66 experimental points (63 + 3 repetitions as described above) with their variables: X_1_: percentage of P80 in P80/SO couple; X_2_: US time; X_3_: proportion of CW in lipidic fraction; X_4_: proportion of GB in lipidic fraction and X_5_: proportion of GDB in lipidic fraction and the responses Y_1_: particle size average (PS), Y_2_: polydispersity index (PDI) and Y_3_: zeta potential (ZP) in absolute value of the SLNs.

Experiences	X_1_	X_2_	X_3_	X_4_	X_5_	Y_1_	Y_2_	Y_3_
>1	0	1	0	0	1	341.8	0.82	39.47
2	0	1	0	1	0	389.6	0.214	45.53
3	0	1	1	0	0	303.1	0.278	47.5
4	0	1	0	0.5	0.5	382.6	0.408	45.43
5	0	1	0.5	0	0.5	354.9	0.31	43.33
6	0	1	0.5	0.5	0	370.3	0.27	37.83
7	0	1	0.33	0.33	0.33	399.9	0.408	40.5
8	50	1	0	0	1	145.8	0.428	33.33
9	50	1	0	1	0	193.1	0.32	28.73
10	50	1	1	0	0	182.9	0.283	34.87
11	50	1	0	0.5	0.5	186.9	0.466	34.23
12	50	1	0.5	0	0.5	208	0.308	31
13	50	1	0.5	0.5	0	197.7	0.248	34.9
14	50	1	0.33	0.33	0.33	241.3	0.31	33.1
15	100	1	0	0	1	244.5	0.488	11.23
16	100	1	0	1	0	272.2	0.121	8.1
17	100	1	1	0	0	83.16	0.543	10.87
18	100	1	0	0.5	0.5	469.9	0.637	12.47
19	100	1	0.5	0	0.5	284.8	0.22	15.1
20	100	1	0.5	0.5	0	21.88	0.43	3.06
21	100	1	0.33	0.33	0.33	24.33	0.537	5.58
22	0	5	0	0	1	215.5	0.388	41.07
23	0	5	0	1	0	245.2	0.279	45.3
24	0	5	1	0	0	243.5	0.27	45.07
25	0	5	0	0.5	0.5	234.8	0.3	41.43
26	0	5	0.5	0	0.5	242.5	0.29	44.9
27	0	5	0.5	0.5	0	245.1	0.23	41.13
28	0	5	0.33	0.33	0.33	261.1	0.248	43.9
29	50	5	0	0	1	117.4	0.346	31.43
30	50	5	0	1	0	122.8	0.45	34.3
31	50	5	1	0	0	106.6	0.299	34.8
32	50	5	0	0.5	0.5	131.6	0.433	31.23
33	50	5	0.5	0	0.5	127.7	0.415	29.67
34	50	5	0.5	0.5	0	150.2	0.244	33.3
35	50	5	0.33	0.33	0.33	159.1	0.26	33.47
36	100	5	0	0	1	203.1	0.4	11.37
37	100	5	0	1	0	452.6	0.295	8.6
38	100	5	1	0	0	252.1	0.22	17.6
39	100	5	0	0.5	0.5	350.3	0.3	9.4
40	100	5	0.5	0	0.5	344.8	0.202	14.93
41	100	5	0.5	0.5	0	25.86	0.646	3.8
42	100	5	0.33	0.33	0.33	29.86	0.604	3.17
43	0	10	0	0	1	225	0.627	39.63
44	0	10	0	1	0	229.3	0.283	40.03
45	0	10	1	0	0	246.1	0.245	42.43
46	0	10	0	0.5	0.5	225.2	0.36	38.97
47	0	10	0.5	0	0.5	249.4	0.368	40.53
48	0	10	0.5	0.5	0	248.7	0.262	41.73
49	0	10	0.33	0.33	0.33	253.9	0.451	38.1
50	50	10	0	0	1	108.7	0.462	32
51	50	10	0	1	0	113.9	0.494	35.87
51′	50	10	0	1	0	111.2	0.41	21.67
52	50	10	1	0	0	98.83	0.384	36.43
52′	50	10	1	0	0	131.2	0.275	32.83
53	50	10	0	0.5	0.5	127.5	0.468	33.53
54	50	10	0.5	0	0.5	125.1	0.399	30.07
55	50	10	0.5	0.5	0	142.3	0.262	33.73
55′	50	10	0.5	0.5	0	128.3	0.307	33.1
56	50	10	0.33	0.33	0.33	160.5	0.296	34.2
57	100	10	0	0	1	287.2	0.89	10.19
58	100	10	0	1	0	712.8	0.292	7.56
59	100	10	1	0	0	208.7	0.37	15.67
60	100	10	0	0.5	0.5	447	0.439	12.7
61	100	10	0.5	0	0.5	316.8	0.251	14.13
62	100	10	0.5	0.5	0	19.44	0.376	4.03
63	100	10	0.33	0.33	0.33	26.07	0.472	3.41

**Table 3:** Type, coefficient, and weight of the responses for the desirability function approach [2].

Response	Type	Min	Max	Target	Weight	Desirability Representation
Size (nm)	Bilateral	50	200	100	1	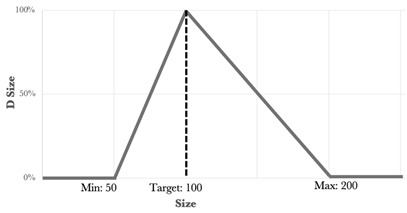
PDI	Right unilateral	-	0.4	0	1	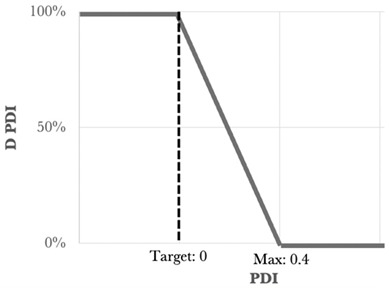
Zeta potential (mV)	Left unilateral	25	-	40	1	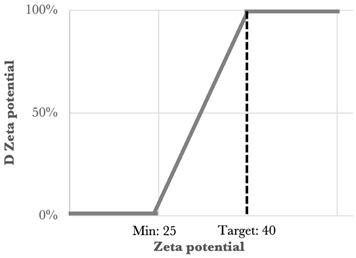

**Table 4:** Coordinates of maximum desirability and observed responses.

Variables	Factors	Values	Responses	Predicted Values	Observed Values
X_1_	% P80	**40.8**	Particle size (nm)	102	**176.3 ± 2.78**
X_2_	Time US	**7.5**	PDI	0.30	**0.268 ± 0.022**
X_3_	CW	**0.59**	Zeta potential (mV)	−34.8	**−35.5 ± 0.36**
X_4_	GB	**0.22**			
X_5_	GDS	**0.19**			

**Table 5:** DSC data of the raw materials and blank SLN.

Samples	N° Peak	Onset (°C)	Peak Temperature (°C)	Enthalpy ∆H (J/g)
CW	1	70.00	82.96	209.74
GB	1	68.02	71.91	151.94
GDS	1	46.42	57.25	135.09
	1 bis		49.39	
Physical mixture of lipids	1	50.76	61.13	36.438
	2	70.47	79.19	52.159
Blank SLN	1	50.10	58.45	10.081
	2	71.08	78.20	18.711

## Data Availability

The original contributions presented in this study are included in the article. Further inquiries can be directed to the corresponding author.
